# Stress Sources and Manifestations in a Nationwide Sample of Pre-Primary, Primary, and Secondary Educators in Greece

**DOI:** 10.3389/fpubh.2016.00073

**Published:** 2016-04-21

**Authors:** Ntina Kourmousi, Evangelos C. Alexopoulos

**Affiliations:** ^1^Directorate of Primary Education of Eastern Attica, Attica, Greece; ^2^School of Social Sciences, Hellenic Open University, Patras, Greece

**Keywords:** teachers’ stress, occupational stress, stress factors, stress manifestations, Teacher Stress Inventory

## Abstract

**Background:**

Teachers experience high levels of stress as a result of their professional duties, and research has shown a growing interest in this phenomenon during the recent years. Aim of this study was to explore the associations of stress sources and manifestations with individual and job-related characteristics in educators of all levels.

**Methods:**

In a cross-sectional design, following an informative e-campaign on the study aims through the official and the main teachers’ portals in Greece, respondents completed online the teachers stress inventory (TSI) and the 14-item Perceived Stress Scale. Nine hundred seventy-four male and 2473 female pre-primary, primary, and secondary educators with a mean age of 41.2 years responded.

**Results:**

Women and younger teachers reported significantly higher levels of stress, mainly due to lack of time and other work-related stressors, and also more emotional and gastronomic manifestations. Increased age and working experience were associated with lower levels of several stress sources. Teachers of administrative positions had increased time management stressors, but less professional distress, professional investment, and discipline and motivation stressors. Additionally, working and residing far from family increased teachers’ stress levels associated with control, motivation, and investment. Teachers of pre-primary education had reduced professional investment and motivation stress factors, while vocational lyceum teachers of secondary education reported less work-related stressors and manifestations and more discipline and motivation-related ones. Having students supported or in need of support from special educators and students with difficulties in speaking or comprehension was associated with most of the teachers’ stress sources and manifestations (i.e., TSI subscales). Finally, colleagues’ and mainly supervisors’ support seemed to provide a strong and consistent protection against both stress sources and manifestations.

**Conclusion:**

Stress factors and manifestations vary among educators by gender, seniority, and teaching level. Training in coping and communication skills starting in teachers’ undergraduate studies might have a major impact on their stress alleviation.

## Introduction

High levels of occupational stress and/or burnout among primary and secondary school teachers have been reported worldwide (1–8). Strangely, most studies conducted in Greece report low levels of stress and/or burnout (9–11), while in Cyprus, higher emotional exhaustion has been reported among regular education teachers compared to their Greek colleagues but not as severe as that of the North Europeans or North Americans (12).

The stress model adapted for educational settings by Kyriakou and Sutcliffe (2) reveals many factors that seem to influence the relationships among occupational stress, job satisfaction, and their consequences (e.g., burnout), including environmental, contextual, and demographic elements and personality characteristics of the teachers (1, 5, 8, 10, 13, 14). Common findings suggest that younger and/or less experienced teachers and females exhibit increased levels of stress and burnout, while males experience a higher level of depersonalization (5, 9, 13, 15, 16). Major factors predicting stress in teaching include time pressure and workload, diversity of job-related tasks, low income, poor prospects of promotion, lack of professional development, and support from school leadership (3, 5, 6, 8, 17–19). Personal and modifiable factors, such as low self-efficacy in classroom management, have also been found to predict emotional exhaustion *via* classroom disturbances (19).

Fimian has largely contributed in monitoring teachers’ stress sources and manifestations by developing the Teacher Stress Inventory (TSI) (20, 21). Other generic stress tools, such as the Perceived Stress Scale (PSS) (22), have been scarcely used in teachers, though they have been used in other groups to assess the effectiveness of stress-reducing interventions (23–29) and the extent of existing associations between psychological stress and psychiatric and physical disorders (30–32).

The impact of various occupational stress factors and its manifestations on teachers of different levels of education, namely, pre-primary, primary, secondary, and even higher education, has not been investigated in extend. This study aims to explore the association of educators’ stress sources and manifestations with individual and job-related characteristics – as tracked by both a specific and a generic stress scale – in a sample of teachers of various teaching levels, in order to contribute to the design of more focused and effective stress management interventions.

## Materials and Methods

### Methodology

In a cross-sectional design, following an informative e-campaign on the study and its aims through main teachers’ portals, educators of different levels from all educational prefectures of Greece voluntarily completed an anonymous online questionnaire that consisted of the TSI and the PSS. The questionnaire was displayed during May 2012 in the International Greek School Network portal www.sch.gr – to which 99.98% of all the pre-primary, elementary, and secondary schools are officially linked – and also in the teachers’ main portal www.alfavita.gr, and in the websites of all teachers’ associations (i.e., www.specialeducation.gr, www.pekade.gr, www.p-e-f.gr, www.inital.gr, etc.), after their affirmative response to our sent request [more study design details have been published elsewhere (33)]. International Greek School Network and Networking Technologies Directorate (IGSN-NTS) gave approval, and informed consent was granted as the participants ticked the corresponding box in the webpage. The Scientific and Ethics committee of the Medical School of Athens’s University approved the conduct of the survey for postgraduate studies.

### Instruments

#### Teacher Stress Inventory

The Fimian TSI (20) consists of 49 questions covering 10 dimensions, divided into stress sources and stress manifestations. Stress factors include five subscales: time management (eight items), work-related stressors (six items), professional distress (five items), discipline and motivation (six items), and professional investment (four items). Stress manifestations include the following subscales: emotional (five items), fatigue (five items), cardiovascular (three items), gastronomic (three items), and behavioral manifestations (four items). Responses are recorded on a 5-point Likert scale and each subscale is scored, one at a time. The minimum and maximum scores that can be achieved on the questionnaire are 49 and 245, respectively. Increased scores in stress sources indicate higher impact of stress factors. Increased scores in stress manifestations indicate greater exhibition of stress-related symptoms and behaviors. The Greek TSI version was validated and found to have satisfactory psychometric properties (33).

#### Perceived Stress Scale

The PSS (22) is the most widely used psychological instrument for assessing the levels of perceived stress. It consists of 14 items that assess the level of stress experienced by the respondent during the previous month, as declared according to the frequency of his/her thoughts and feelings over the occurrence of events and situations. The 14-item Perceived Stress Scale (PSS-14) responses range from 0 (never) to 4 (very often), while the total scoring ranges from 0 to 56 and comes from reversing the scores of four positive items and then summing up all 14 items. High scores on the PSS-14 indicate high stress levels, while low ones indicate less stress. The Greek PSS version that was used in the present study has been validated and found to have satisfactory psychometric properties (34).

### Statistical Analysis

Continuous variables are presented with mean and SD, while quantitative variables are presented with absolute and relative frequencies. Multiple linear regression analyses were conducted in order to explore the association of TSI subscales and PSS with demographics and work-related characteristics. The regression equation included terms for gender, age, educational level (pre-primary, primary, secondary, and higher education), position (administrative or not), years of previous employment, working in the public or private sector, job status (permanent or substitute), residing far from family due to workplace, number of students in the class, working in special education, presence of students supported or in need of support from special educators according to expert advice or personal opinion, presence of students with difficulties in speaking or comprehension, and also for existence of support from colleagues or supervisors. Teachers’ willingness to voluntarily attend a free educational program in stress management was also explored. Regression coefficients and SE were computed from the results of the linear regression analyses. Diagnostics for regression models were executed in order to check if the conditions for regression had been met with the residuals of each model being normally distributed and their variance being constant. Hypothesized interactions of variables in the models were not significant. All reported *p*-values are two-tailed. Statistical significance was set at *p* < 0.01 and analyses were conducted by the use of SPSS statistical software (version 19.0).

## Results

Sample consisted of 3447 participants (28.3% men and 71.7% women) with mean age of 41.2 years (SD = 8.7 years). Sample characteristics are shown in Table 1. Most of the participants worked in the public sector (88.2%) and had a permanent job (87.9%), while 11.1% of the teachers had an administrative position (director). About 15.5% of them resided far from their family due to their workplace. About 61.9% of teachers had a bachelor (4-year university attendance) degree, 31.3% had in addition an MSc or a PhD diploma, and 6.8% of them had other kind of degrees or diplomas (i.e., from public or private 2-year colleges). More than 50% of them taught in secondary schools, namely, high schools (first 3 years of secondary education) and general and vocational lyceums (second 3 years of secondary education), while 31% taught in primary education and 10.3% in preschool education. About 5.5% of the sample worked in special education. The proportion of participants who reported having support from their colleagues and from their supervisors was 92 and 78.9%, respectively. The majority of the participants (83%) declared that they would voluntarily attend an educational course in stress management that would last more than 2 days.

**Table 1:** **Job characteristics of the 3447 (974 males/2473 females) educators**.

	*N*	%
Teaching in		
Preschool (pre-primary)	355	10.3
Primary	1069	31
Lower secondary (first 3 years of high school)	793	23
Upper secondary (lyceum, next 3 years of high school)	686	19.9
Vocational lyceum (next 3 years of high school)	295	8.6
Other schools (foreign language, cram schools)	249	7.2
Teaching in special education	189	5.5
Public/private sector	3039/408	88.2/11.8
Number of students in the class, mean (SD)	20.7	11.8
Student(s) supported or in need of support from special educators, based on		
Expert advice	1816	52.7
Teacher opinion	2327	67.5
Student(s) with difficulties in speaking or comprehension	1975	57.3
Residing away from family, due to work	534	15.5
Lack of support by colleagues	275	8.0
Lack of support by supervisors	726	21.1
Willing to attend a stress management course (>2 days long)	2860	83.0

### Stress Factors

Multiple linear regression analyses for TSI sources subscales (Table 2) showed that female teachers had increased time management and work-related stressors, while age was negatively correlated with time management and work-related stress factors, as with professional distress and professional investment ones. Increased years of previous employment were associated with lower professional distress and with reduced discipline and motivation stress factors. Residing away from their family due to workplace unfavorably affected the teachers’ levels of discipline and motivation and professional investment stressors. Additionally, teachers of administrative positions (school directors and heads of kindergartens) had worst scores on time management but had fewer professional distress, discipline and motivation, and professional investment stress factors than their subordinates.

**Table 2:** **Multiple linear regression analyses for stress factors (subscales: time management, work-related stressors, professional distress, discipline and motivation, and professional investment) (all analyses are adjusted for educational category)**.

	Time management		Work-related stressors		Professional distress		Discipline and motivation		Professional investment	
Mean (SD)	3.0	(0.8)	2.8	(0.9)	3.1	(1.1)	2.7	(1.0)	2.3	(1.0)
	**β (SE)^a^**	***p***	**β (SE)**	***p***	**β (SE)**	***p***	**β (SE)**	***p***	**β (SE)**	***p***
Gender (RC: males)										
Females	0.22 (0.03)	<0.001	0.21 (0.04)	<0.001	−0.08 (0.04)	0.065	0.05 (0.04)	0.201	−0.06 (0.04)	0.106
Age	−0.007 (0.003)	0.026	−0.010 (0.003)	0.004	−0.021 (0.004)	<0.001	−0.003 (0.004)	0.496	−0.009 (0.004)	0.017
Degree (RC: MSc/PhD)										
University (BSc)	−0.14 (0.03)	<0.001	−0.13 (0.03)	<0.001	−0.08 (0.04)	0.056	0.03 (0.04)	0.411	−0.05 (0.04)	0.171
Years of employment	0.002 (0.001)	0.611	0.004 (0.003)	0.280	−0.011 (0.004)	0.004	−0.011 (0.004)	0.002	−0.004 (0.004)	0.226
Sector (RC: public)										
Private	0.19 (0.11)	0.070	0.17 (0.11)	0.143	−0.5 (0.13)	0.001	−0.22 (0.13)	0.078	−0.16 (0.12)	0.547
Administrative position	0.14 (0.05)	0.001	0.28 (0.05)	0.102	−0.23 (0.06)	<0.001	−0.33 (0.05)	<0.001	−0.24 (0.05)	<0.001
Contract (RC: permanent)										
Substitute	−0.09 (0.05)	0.081	−0.07 (0.05)	0.159	−0.18 (0.07)	0.005	−0.10 (0.06)	0.116	−0.1 (0.06)	0.106
Number of students in class	0.002 (0.003)	0.101	0.010 (0.001)	<0.001	0.002 (0.002)	0.327	0.002 (0.002)	0.278	0.002 (0.001)	0.350
Working in special education (RC: no)	0.04 (0.07)	0.563	−0.05 (0.07)	0.481	−0.11 (0.08)	0.196	−0.32 (0.08)	<0.001	0.09 (0.08)	0.501
Students in class, who are supported or need support from special educators (RC: no)										
Expert advice	0.01 (0.03)	0.773	0.07 (0.04)	0.056	0.07 (0.04)	0.119	0.09 (0.04)	0.023	0.05 (0.04)	0.750
Personal opinion	0.11 (0.04)	0.002	0.12 (0.04)	0.003	0.14 (0.05)	0.004	0.20 (0.04)	<0.001	0.13 (0.04)	0.148
Students with difficulties in speaking or comprehension (RC: no)	0.1 (0.03)	0.001	0.16 (0.03)	<0.001	0.14 (0.04)	<0.001	0.08 (0.04)	0.039	0.06 (0.04)	0.760
Residing away from family due to work (RC: no)	−0.05 (0.04)	0.155	−0.03 (0.04)	0.451	0.09 (0.05)	0.081	0.14 (0.05)	0.005	0.12 (0.05)	0.077
Colleagues’ support (RC: no)	−0.14 (0.06)	0.015	−0.11 (0.06)	0.079	−0.35 (0.07)	<0.001	−0.1 (0.07)	0.150	−0.43 (0.07)	0.024
Supervisors’ support (RC: no)	−0.15 (0.04)	<0.001	−0.19 (0.04)	<0.001	−0.38 (0.05)	<0.001	−0.24 (0.04)	<0.001	−0.48 (0.04)	0.008

*RC, reference category*.

*^a^Regression coefficients (β) and SE*.

Teachers with a university degree had significantly reduced time management and work-related stressors, compared to those with an MSc degree and/or a PhD degree. Professional distress was lower for teachers working in private organizations compared to those working in public ones, as it was also lower for substitute teachers compared to those with permanent positions.

Teachers of general lyceum (the last 3 years of secondary education for students aiming to proceed to higher education) and “other” (e.g., private or public higher education educators and professors) had increased work-related stressors compared to those teaching in vocational lyceum (the last 3 years of secondary education for students aiming to go straight to the job market). Also, teachers of vocational lyceum had greater levels of discipline and motivation stressors compared to those teaching in preschool and primary education, in high school (the first 3 years of secondary education), and in general lyceum. Additionally, teachers of primary education, high school, general lyceum, and vocational lyceum had more professional investment stressors compared to those teaching in preschool education (data not shown in tables).

Increased number of students in the class appeared related to increased work-related stressors, and working in special education was found associated with greater stress relative to discipline and motivation. Furthermore, the existence of classroom students supported or in need of support from special education teachers, according to expert advice, was associated with more discipline and motivation stressors, while the existence of classroom students supported or in need of support from special education teachers according to participants’ personal opinion was associated with greater impact of all TSI stress factors. Also, the existence of classroom students with difficulties in speaking or comprehension was associated with increased time management, work-related, professional distress, and discipline and motivation-related stress.

Finally, support from colleagues or supervisors was found to have a protective effect on many stress domains. Specifically, support from colleagues was associated with less time management, professional distress, and professional investment stress factors, while support from supervisors was associated with lower levels of all TSI stress sources.

### Stress Manifestations

Multiple linear regression analyses for TSI manifestations subscales (Table 3) showed that female teachers exhibited more stress manifestations on all dimensions except for the behavioral ones, compared to their male counterparts. Age was independently and negatively correlated with emotional and gastronomic manifestations subscales. Increased years of previous employment were associated not only with less emotional and fatigue manifestations but also with more gastronomic and behavioral ones. Also, teachers of administrative positions reported more behavioral manifestations.

**Table 3:** **Multiple linear regression analyses for stress manifestations (subscales: emotional, fatigue, cardiovascular, gastronomic, and behavioral) (all analyses are adjusted for educational category)**.

	Emotional		Fatigue		Cardiovascular		Gastronomic		Behavioral	
Mean (SD)	2.8	(1.1)	2.7	(1.0)	2.7	(1.2)	2.1	(1.1)	1.3	(0.5)
	**β (SE)^a^**	***p***	**β (SE)**	***p***	**β (SE)**	***p***	**β (SE)**	***p***	**β (SE)**	***p***
Gender (RC: males)										
Females	0.44 (0.05)	<0.001	0.35 (0.04)	<0.001	0.29 (0.05)	<0.001	0.29 (0.05)	<0.001	−0.03 (0.02)	0.215
Age	−0.010 (0.004)	0.018	0.001 (0.004)	0.956	0.001 (0.005)	0.759	−0.011 (0.005)	0.018	0.001 (0.002)	0.748
Degree (RC: MSc/PhD)										
University (BSc)	0.10 (0.04)	0.017	0.06 (0.04)	0.136	0.19 (0.05)	<0.001	0.04 (0.05)	0.337	−0.08 (0.04)	0.026
Years of employment	−0.008 (0.004)	0.048	−0.008 (0.004)	0.038	0.003 (0.004)	0.522	0.009 (0.031)	0.031	0.006 (0.002)	0.001
Sector (RC: public)										
Private	0.01 (0.14)	0.952	−0.02 (0.13)	0.907	0.09 (0.16)	0.547	−0.04 (0.15)	0.792	−0.02 (0.03)	0.569
Administrative position	−0.05 (0.06)	0432	−0.04 (0.06)	0454	0.30 (0.07)	0.639	0.11 (0.06)	0.081	0.07 (0.03)	0.017
Contract (RC: permanent)										
Substitute	−0.01 (0.07)	0.904	−0.04 (0.07)	0.524	−0.05 (0.08)	0.507	0.04 (0.07)	0.581	0.00 (0.03)	0.961
Number of students in class	0.001 (0.002)	0.920	0.002 (0.002)	0.234	0.002 (0.002)	0.350	0.001 (0.002)	0.652	0.002 (0.001)	0.024
Working in special education (RC: no)	0.19 (0.09)	0.033	0.17 (0.08)	0.043	0.07 (0.10)	0.501	−0.02 (0.09)	0.868	0.05 (0.04)	0.262
Students in class, who are supported or need support from special educators (RC: no)										
Expert advice	−0.06 (0.05)	0.215	0.03 (0.04)	0.518	−0.02 (0.05)	0.750	−0.01 (0.05)	0.806	0.00 (0.02)	0.856
Personal opinion	0.12 (0.05)	0.018	0.11 (0.05)	0.021	0.08 (0.06)	0.148	0.13 (0.05)	0.018	0.01 (0.02)	0.528
Students with difficulties in speaking or comprehension (RC: no)	0.04 (0.04)	0.312	0.01 (0.04)	0.707	0.01 (0.05)	0.760	0.08 (0.04)	0.054	0.02 (0.02)	0.238
Residing away from family due to work (RC: no)	0.06 (0.06)	0.300	0.08 (0.05)	0.126	0.10 (0.06)	0.077	0.07 (0.06)	0.271	0.03 (0.02)	0.254
Colleagues’ support (RC: no)	−0.09 (0.08)	0.272	−0.32 (0.07)	<0.001	−0.20 (0.09)	0.024	−0.02 (0.08)	0.811	−0.06 (0.04)	0.125
Supervisors’ support (RC: no)	−0.24 (0.05)	<0.001	−0.24 (0.05)	<0.001	−0.15 (0.05)	0.008	−0.21 (0.05)	<0.001	−0.10 (0.02)	<0.001

*RC, reference category*.

*^a^Regression coefficients (β) and SE*.

Teachers with a university degree had significantly increased emotional and cardiovascular stress manifestations compared to those with an MSc degree and/or a PhD degree, while higher educated participants had reduced behavioral stress exhibition.

Fatigue-related stress manifestations appeared elevated for educators teaching in high school and general lyceum compared to those teaching in vocational lyceum, while more cardiovascular manifestations were reported by teachers working in primary education compared to those teaching in general lyceum (data not shown in tables).

Increased number of classroom students was found to increase teachers’ behavioral manifestations, while working in special education was found associated with more emotional and fatigue stress manifestations. Furthermore, the existence of classroom students supported or in need of support from special educators, according to participant’s opinion, was associated with more emotional, fatigue, and gastronomic stress manifestations.

Support from colleagues was associated with less fatigue and cardiovascular stress manifestations in teachers, while support from supervisors was associated with lower scores on all TSI stress manifestation subscales.

### Perceived Stress Scale and TSI Total Score

Multiple linear regression analyses for the PSS scale (Table 4) showed that women and teachers with students who had difficulties in speaking or comprehension reported higher levels of perceived stress. Longer employment was associated with lower scores on the PSS and support from colleagues and supervisors had a protective effect on teachers’ stress levels as assessed by the PSS.

**Table 4:** **Multiple linear regression analyses for the TSI and PSS total score (all analyses are adjusted for educational category)**.

	TSI		PSS	
	β (SE)^a^	*p*	β (SE)	*p*
Gender (RC: males)				
Females	0.15 (0.03)	<0.001	2.15 (0.34)	<0.001
Age	−0.01 (0.001)	<0.001	0.017 (0.032)	0.018
Degree (RC: MSc/PhD)				
University (BSc)	−0.01 (0.02)	0.586	−0.49 (0.33)	0.136
Years of employment	−0.004 (0.002)	0.066	−0.065 (0.031)	0.035
Sector (RC: public)				
Private	0.03 (0.04)	0.522	−0.57 (1.08)	0.597
Administrative position	−0.03 (0.04)	0.463	−0.84 (0.45)	0.067
Contract (RC: permanent)				
Substitute	−0.07 (0.06)	0.259	−0.67 (0.53)	0.205
Number of students in class	0.003 (0.001)	0.006	0.010 (0.013)	0.422
Working in special education (RC: no)	−0.003 (0.05)	0.958	0.56 (0.68)	0.407
Students in class, who are supported or need support from special educators (RC: no)				
Expert advice	0.01 (0.03)	0.644	0.53 (0.36)	0.133
Personal opinion	0.13 (0.03)	<0.001	0.57 (0.38)	0.136
Students with difficulties in speaking or comprehension (RC: no)	0.07 (0.02)	0.003	1.14 (0.31)	<0.001
Teaching and living away from family (RC: no)	0.06 (0.03)	0.043	0.44 (0.42)	0.294
Colleagues’ support (RC: no)	−0.19 (0.05)	<0.001	−2.49 (0.60)	<0.001
Supervisors’ support (RC: no)	−0.24 (0.03)	<0.001	−2.62 (0.38)	<0.001

*RC, reference category*.

*^a^Regression coefficients (β) and SE*.

Women, younger participants, and teachers with an increased number of students in their class also had greater TSI total scores. Additionally, conditions, such as residing far from family, lacking support from colleagues and supervisors, having students in need of special education according to own opinion, or students with difficulty in speaking or understanding Greek, were independently associated with greater total stress levels.

It is worth mentioning that teachers who would voluntarily attend a free educational program in stress management, which would last more than 2 days, had worst scores on all TSI stress factors subscales, on all TSI stress manifestations subscales, and on PSS.

## Discussion

Stress in teaching is a well-recognized phenomenon affected by various factors, including personal and job-related characteristics (1–8, 10–15, 35). In our study, we used the TSI and the PSS to explore associations between such factors and teachers’ stress in a large sample of educators of different teaching levels in Greece.

Female teachers were found to have higher levels of work-related and perceived stress. Other researchers have reported similar findings (36, 37), although evidence of no significant gender differentiations regarding occupational stress has also been published (38, 39). Our findings may be explained by differences in psychological and personality traits, such as self-esteem (40), differences in skills, such as problem-solving strategies (41), and differences in roles and role expectations (42), since as reported, women get more affected by family–job conflicts (43).

Age and seniority were inversely related to teachers’ stress levels; the elder the teachers were, the less they were affected by work-related stress factors and time management, professional distress, and professional investment stressors. In analogy, longer employment was associated with reduced professional distress and discipline and motivation stressors and less perceived stress (PSS), as also reported by other studies (5, 9). This finding could be explained by the fact that long-term teaching experience provides teachers with strength and resilience.

As expected, working and residing far from the family influenced teachers’ professional investment unfavorably, also increasing their discipline and motivation stressors. This practice is very common in Greece for younger and newly appointed teachers during the first years of their career, so age and inexperience may hold an influence. This finding also reveals the necessity to focus on stress management and empowerment training early in teachers’ careers, starting with their under graduate studies.

Another interesting finding was that teachers with postgraduate studies had elevated time management and work-related stressors. This probably occurs due to the increased obligations and/or expectations of these individuals – by both their superiors and themselves – and thus to the extra time and effort needed in order to fulfill them. It seems of importance to embody stress management courses in teachers’ postgraduate programs as well.

The finding that professional distress, discipline and motivation, and professional investment did not constitute stressors for teachers of administrative positions who seemed to be pressured only by time management did not surprise us; school directors usually have a lot to deal with – paperwork, parents–teacher issues, organizational matters, etc. – but they also have strong beliefs of having evolved in their profession and of being capable to enforce order in their schools.

Professional distress was lower for teachers working in private organizations compared to those working in public ones, as it was also lower for substitute teachers compared to those with permanent positions. These findings were somewhat troubling to interpret at first, given that a permanent job provides more security. One possible cause could be the continuous changes and reforms imposed by the Greek educational authorities that put a lot of pressure and create a climate of professional instability for permanent public teachers, as stated earlier by Kantas (43). Additionally, Pomaki and Anagnostopoulou (44) found that the permanent employment status, combined with the structure of academic education and the lack of teachers’ assessment, makes the teaching profession in Greece repetitive and monotonous and may contribute in lack of motivation, thus possibly leading to lower self-perceptions.

Our study also confirmed the findings of other researchers who reported that special education teachers usually have a higher degree of job-related stress than the regular education ones (18, 35, 45–47), contrary to the study of Sutton and Huberty (48) who found no significant difference between the two educator groups, and the one of Nagel and Brown (49) who found special education teachers to be less stressed. Among the relatively stable findings in special education teachers is that those working with children with multiple disabilities and learning difficulties, consistently report lower levels of stress, in comparison with those who work with students with emotional and behavioral disorders and students who have poor motivation (22, 47, 50, 51).

Teachers of pre-primary education had reduced professional investment and motivation stressors. This could be explained by the fact that there is no strict curriculum to be followed in Greek preschool education, and supervisors’ and parents’ demands from kindergarten teachers are fewer than those required of other educational level teachers. It seems that preschool teachers feel a sense of control over their work and have more time for improvement and for emotional and intellectual stimulation. Several studies support the finding that lack of control over the teaching context and procedure increases educators’ stress (44, 52).

Vocational lyceum teachers had fewer work-related stressors and manifestations but more discipline and motivation ones. This can be attributed to the fact that these teachers do not have a large work load as their colleagues of general lyceums – since their students are not intended to attend higher education – but they do face problems concerning classroom control and providing adolescents with incentives.

As with stress factors, female teachers reported more stress manifestations, on all (emotional, fatigue, cardiovascular, and gastronomic) but the behavioral TSI subscale. Other researchers have also reported that women cope differently from men in problem situations and that they are more susceptible to emotional exhaustion and more easily affected by negative emotions (9, 14, 17, 36, 41). Less emotional stress manifestations were also recorded in elders and in those with longer working experience, implying that experience gained by age and training mediates and affects the cognitive appraisal of a job stressor, thus making coping easier. Besides, experience helps teachers feel more adapted to school working conditions and environment (36).

More fatigue was reported by educators of general secondary education (high school and general lyceum), which may be explained by the workload and the pressure they undertake, in order to meet the expectations for adequate preparation of students for university admission. By contrast, vocational lyceum teachers face no such pressure, since their graduate students proceed straight to the job market. Our conclusion is supported by the finding that high job demands predict low personal accomplishment, probably due to lack of time to complete tasks, as assumed by Pomaki and Anagnostopoulou (44).

Increased emotional and fatigue manifestations reported by teachers working in special education and by those with students supported or in need of support from special educators were anticipated, since working with children with special educational needs has been recognized to create extra pressure for teachers (12, 18). Equally reasonably, the number of classroom students appeared to affect the behavioral stress manifestations of educators, a fact that could be attributed not only to the large workload but also to the difficulty to coordinate the class and retain control of it.

Finally, we confirm that support from colleagues and supervisors has a protective effect for teachers on many stress domains (44, 52, 53). Interestingly, supervisors’ support seems to contribute more than colleagues’ support in stress manifestations’ reduction; colleagues’ support was found to reduce teachers’ fatigue and cardiovascular manifestations, while supervisors’ support was found to reduce all TSI stress manifestations. After all, support has a well-known protective effect in various occupational groups (6, 44, 52, 54). It seems that getting help from other individuals in the workplace when needed and feeling supported is of importance to teachers, and could serve as a strong protective buffer against occupational stress (55, 56).

The fact that the vast majority of teachers who reported that they would voluntarily attend a stress management program was related to increased stress manifestations in all TSI subscales, indicates that they have taken the first step in identifying their problem, and also that they are prepared to take actions in order to deal with it.

## Conclusion and Implications

In our study, stress factors and manifestations were found to vary by teaching level, seniority, and job status. Female, younger, and inexperienced teachers, along with those having students with difficulties, reported increased stress sources and manifestations. At the same time, colleagues’ and mainly supervisors’ support seemed to provide strong protection against both stress sources and manifestations.

Our findings underline the need (a) to design appropriate and specialized stress management training for teachers early in their career, taking into account the educational level they will teach in and the relevant job demands, (b) to include communication and counseling skills in their training in order to enhance future supporting working environments, and (c) to systematically monitor factors, such as difficult students’ situations, in order to design effective interventions.

Stress in teaching cannot be eliminated. It could, however, be reduced to manageable levels in order for teachers to be able to function efficiently and maintain mental health and inner harmony. Providing educators with training on how to maintain a balance between work demands and duties, and personal and social-life time, on how to set realistic targets in order to fulfill them, on how to communicate and sustain supportive relations in the workplace, and on how to develop stress-coping strategies, should be foreseen and implemented in undergraduate and postgraduate teachers’ studies. Further studies should be conducted, assessing various forms of such training, in order to produce evidence-based training interventions, appropriate for educators of each teaching level.

Building supportive and helpful relations in the workplace and practicing coping and empowering techniques may help teachers significantly in reducing anxiety and in dealing with occupational stress.

### Strengths and Limitations

The main strength of the current study is the large sample size of teachers and the inclusion of educators of all teaching levels and positions. Having in mind that teachers’ voluntary participation in our study could possibly influence our results – as is usual in all cross-sectional surveys – we need to stress that our sample came from addressing the whole Greek teachers’ population, since the study was conducted mainly through the International Greek School Network portal, to which 99.98% of schools from all over Greece are officially linked. The ratios of categories in our research regarding educators’ population, namely, their age, sex, and teaching level, are in line with those provided by the Greek Statistical Office for the specific school year the study was conducted (57).

However, the cross-sectional nature of our investigation does not allow reaching absolute causal interpretations regarding the association between job dimensions and variables of stress factors and manifestations. Furthermore, personality measures were not included in our research design and, therefore, the intermediate role of these variables cannot be considered.

## Author Contributions

NK and EA designed the study protocol and organized the survey. NK managed and co-coordinated the data collection, participated in the analysis, and in drafting and revising the manuscript. EA supervised the design and conduct of the study, participated in analysis, and in drafting and revising the manuscript. Both authors read and approved the final manuscript.

## Conflict of Interest Statement

The authors declare that the research was conducted in the absence of any commercial or financial relationships that could be construed as a potential conflict of interest.

## Abbreviations

PSS, Perceived Stress Scale; PSS-14, 14-item Perceived Stress Scale; TSI, Teachers Stress Inventory.
